# Simple and Efficient Synthesis of Ruthenium(III) PEDOT:PSS Complexes for High-Performance Stretchable and Transparent Supercapacitors

**DOI:** 10.3390/nano14100866

**Published:** 2024-05-16

**Authors:** Guiming Liu, Zhao Huang, Jiujie Xu, Bowen Zhang, Tiesong Lin, Peng He

**Affiliations:** 1 State Key Laboratory of Precision Welding & Joining of Materials and Structures, Harbin Institute of Technology, Harbin 150001, China; liu.guiming@outlook.com (G.L.); huangzhaohit@outlook.com (Z.H.); 20b909121@stu.hit.edu.cn (J.X.); 2 School of Electrical Engineering, Tiangong University, Tianjin 300350, China; bowenzhang@tju.edu.cn

**Keywords:** supercapacitors, ruthenium, PEDOT:PSS, complexes, stretchable, transparent

## Abstract

In the evolving landscape of portable electronics, there is a critical demand for components that meld stretchability with optical transparency, especially in supercapacitors. Traditional materials fall short in harmonizing conductivity, stretchability, transparency, and capacity. Although poly(3,4-ethylenedioxythiophene):poly(styrene sulfonate) (PEDOT:PSS) stands out as an exemplary candidate, further performance enhancements are necessary to meet the demands of practical applications. This study presents an innovative and effective method for enhancing electrochemical properties by homogeneously incorporating Ru(III) into PEDOT:PSS. These Ru(III) PEDOT:PSS complexes are readily synthesized by dipping PEDOT:PSS films in RuCl_3_ solution for no longer than one minute, leveraging the high specific capacitance of Ru(III) while minimizing interference with transmittance. The supercapacitor made with this Ru(III) PEDOT:PSS complex demonstrated an areal capacitance of 1.62 mF cm^−2^ at a transmittance of 73.5%, which was 155% higher than that of the supercapacitor made with PEDOT:PSS under comparable transparency. Notably, the supercapacitor retained 87.8% of its initial capacitance even under 20% tensile strain across 20,000 cycles. This work presents a blueprint for developing stretchable and transparent supercapacitors, marking a significant stride toward next-generation wearable electronics.

## 1. Introduction

The rapid advancement in portable intelligent electronics necessitates the development of devices that combine stretchability with optical transparency [1,2,3]. This requirement extends to a wide array of applications, including but not limited to displays, panels, smartphones, tablets, and other interactive devices [4,5,6]. Consequently, it is imperative that all essential components, notably solar cells, supercapacitors, batteries, sensors, and displays, also embody these attributes of stretchability and transparency [7,8,9,10].

In the realm of energy conversion/storage solutions, supercapacitors emerge as a preferable choice, owing to their outstanding power density, rapid charging and discharging capabilities, maintenance-free operation, and longevity [11,12,13]. Moreover, compared to traditional batteries, supercapacitors offer considerable advantages in terms of portability and safety. This is attributed to their lighter weight and lower risk of explosion, an essential consideration given that these devices are often designed for wearability and exposure to constant mechanical stressors, including bending, twisting, and stretching [14,15,16]. In contrast to batteries, whose charge rates are kinetically constrained, supercapacitors boast much higher charge and discharge rates. The cycle life of supercapacitors (more than 50,000 h) is remarkably higher than that of batteries, and supercapacitors offer remarkably short recharge times (supercapacitor: 1–10 s vs. battery: 1–10 h) [17,18]. However, achieving high capacity, conductivity, stretchability, and transparency in supercapacitors presents a significant challenge. Most conventional electrode materials, including metal oxides and carbon materials, fail to meet these comprehensive criteria.

To address the challenges presented by conventional materials, poly(3,4-ethylenedioxythiophene):poly(styrene sulfonate) (PEDOT:PSS) has emerged as a promising alternative. It facilitates the fabrication of supercapacitors that meet the requirements for stretchability and transparency [19]. PEDOT:PSS is distinguished by its ultra-high intrinsic stretchability, achieving tensile strains of over 100% once doped. It also features high transparency (over 90%), high conductivity (over 4000 S cm ^−1^), and relatively high specific capacitance (1.18 mF cm^−2^) [20,21]. Additionally, it offers electrochemical stability and is solution-processable [22,23]. Several groups have reported the fabrication of transparent supercapacitors based on PEDOT:PSS. For example, Higgins et al. and Cheng et al. fabricated transparent and flexible PEDOT:PSS supercapacitor electrodes through spray-depositing or spin-coating its solution onto a PET substrate [24,25]. The PEDOT:PSS film was used as both active materials and current collectors. Sequentially, researchers have focused on developing optimal capacitance devices by incorporating PEDOT:PSS with high-capacity pseudocapacitive materials. Huang et al. reported flexible transparent supercapacitors comprising Ag nanowires/Na_x_WO_3_ nanowires/PEDOT:PSS/PET anode, PEDOT:PSS/PET cathode, and poly(acrylic acid)/H_2_SO_4_ gel electrolyte [26]. The asymmetric supercapacitor displayed a specific capacitance of 1.109 mF cm^−2^, although the transmittance in the visible light region was only 55%. Among the pseudocapacitive materials, the Ru compound has been the most extensively studied candidate due to its high specific capacitance (about 1000 F g^−1^), wide potential window (1.2 V), highly reversible redox reactions, high proton conductivity, good thermal stability, long cycle life, and high rate capability [27]. These advantages make Ru compound a suitable candidate material for incorporating with PEDOT:PSS. Zhang et al. created transparent and flexible PEDOT:PSS/RuO_2_ supercapacitor electrodes through aerosol-jet spraying a mixed solution of PEDOT:PSS and RuO_2_ nanoparticles [28]. The assembled symmetric supercapacitors exhibited 80% transparency and an areal capacitance of 0.84 mF cm^−2^, 73.7% higher than PEDOT:PSS supercapacitors. However, pseudocapacitive materials in these studies were synthesized by the hydrothermal method with high-temperature and high-pressure, with disadvantages such as complicated operation, long synthesis period, and high manufacturing cost. Additionally, these supercapacitors are merely flexible rather than fully utilizing the intrinsic high stretchability of PEDOT:PSS.

In this investigation, we elucidate a simple and efficient methodology to prepare Ru(III) PEDOT:PSS complexes as electrode material for stretchable and transparent supercapacitors. The complexes are readily synthesized by dipping PEDOT:PSS films in RuCl_3_ solution for no longer than one minute. This process eliminates the need for elevated temperatures or pressures. The structure of the complexes was systematically investigated, with a detailed examination of the influence of immersion duration on electrode efficacy, including transmittance and electrochemical attributes. The functional applicability of these Ru(III) PEDOT:PSS complexes was confirmed via the assembly of symmetric solid-state supercapacitors. This assembly involved the integration of the electrodes with a lithium chloride/poly(vinyl alcohol) (LiCl/PVA) gel electrolyte, culminating in a specific capacitance of 1.62 mF cm^−2^ at a transmittance of 73.5%. Notably, this capacitance exceeds the baseline performance of supercapacitors fabricated with PEDOT:PSS by 155% under comparable transparency. Furthermore, the electrode exhibited a retention of 93.6% of its initial capacitance after 5000 charge/discharge cycles, with the device maintaining 87% of its initial capacitance even under a 20% tensile strain across 20,000 cycles, evidencing superior electrochemical resilience and stretchable durability. This research not only introduces a new class of Ru(III) complexes but also outlines an innovative material for creating stretchable, transparent supercapacitors. It marks significant advancements for the next generation of portable and intelligent electronic devices.

## 2. Materials and Methods

### 2.1. Materials

Polydimethylsiloxane (PDMS) (Sylgard 184) was purchased from Dow Corning (Midland, MI, USA). Capstone FS-3100 was purchased from DuPont (Wilmington, DE, USA). Ethylene glycol (EG), Triton X-100, poly(vinyl alcohol) 1788 (PVA 1788), ruthenium chloride hydrate (RuCl_3_·nH_2_O), and lithium chloride (LiCl) were purchased from Aladdin (Shanghai, China) and used as received. PEDOT:PSS (Clevios PH1000) aqueous dispersion was purchased from Heraeus and filtered with 0.22 μm filters before use. The required amount of RuCl_3_·nH_2_O was dissolved in deionized water to provide the desired concentration. Given that RuCl_3_ is classified as a hazardous material due to its corrosive and toxic properties, researchers should wear appropriate personal protective equipment, including gloves, safety goggles, and lab coats, to prevent direct contact with RuCl_3_ and minimize exposure risks. Additionally, waste containing RuCl_3_ should be collected and disposed of in accordance with local regulations and institutional guidelines to facilitate safe handling and disposal.

### 2.2. Fabrication of PEDOT:PSS and Ru(III) PEDOT:PSS Complex Films

The original PEDOT:PSS dispersion was first spin-coated or drop-casted on the glass substrate. It was then dried on the hot plate at 70 °C for 20 min and cooled to room temperature, forming PEDOT:PSS film with glass. For the Ru(III) PEDOT:PSS complex film, glass-supported PEDOT:PSS film was further dipped in 100 mmol RuCl_3_ solution for 1 min to ensure the complete transformation of PEDOT in Ru(III) PEDOT:PSS complexes. This process occurs spontaneously and rapidly at room temperature, without the need for elevated temperature or pressure, as well as without stirring or the use of catalysts, as is also the case in the following sections. Residual RuCl_3_ was removed by immersing the glass-supported film in deionized water. Finally, the glass-supported Ru(III) PEDOT:PSS complex film was obtained by drying in air.

### 2.3. Fabrication of PEDOT:PSS and Ru(III) PEDOT:PSS Complex Electrodes

The original PEDOT:PSS solution was mixed with 2 wt% Triton X-100 and 5 wt% EG. The well mixed PEDOT:PSS dispersion was spin-coated onto glass substrate with different spin speeds. It was then dried on the hot plate at 70 °C for 20 min and cooled to room temperature, forming the PEDOT:PSS electrode. For the Ru(III) PEDOT:PSS complex electrodes, glass-supported PEDOT:PSS film was further dipped in 2 mmol RuCl_3_ solution for 10 to 60 s, then immersed in deionized water to remove residual RuCl_3_. These electrodes were painted with silver paste and attached to copper tape before electrochemical characterization.

### 2.4. Fabrication of PEDOT:PSS and Ru(III) PEDOT:PSS Complex Supercapacitors

The base and curing agent of the PDMS (10:1, weight ratio) was mixed in a beaker. This PDMS mixture was poured onto a glass plate and cured at 70 °C for 2 h, forming a PDMS/glass substrate. The original PEDOT:PSS dispersion was mixed with 2 wt% Triton X-100, 5 wt% EG, and 15 wt% Capstone FS-3100. The well-mixed PEDOT:PSS solution was spin-coated onto the PDMS/glass substrate. It was then dried on a hot plate at 70 °C for 20 min and cooled to room temperature, forming the PEDOT:PSS transparent and stretchable electrode. To fabricate Ru(III) PEDOT:PSS complex transparent and stretchable electrodes, the PDMS/glass-supported PEDOT:PSS film was further dipped in a 2 mmol RuCl_3_ solution for 40 s, followed by immersion in deionized water to remove residual RuCl_3_. These glass-supported transparent and stretchable electrodes were painted with silver paste and attached to copper tape, and then these positions were sealed with PMDS. The LiCl/PVA gel electrolyte was prepared by mixing PVA 1788 (5.0 g), LiCl (5.0 g), and deionized water (50.0 g). The mixture was stirred at room temperature until it became a clean gel. Subsequently, PEDOT:PSS or Ru (III) PEDOT:PSS complex film with the PDMS substrate was peeled off from the glass. These stretchable electrodes were coated with LiCl/PVA gel electrolyte and dried in air for 5 h. Finally, two identical electrodes were assembled into a supercapacitor by pressing them together.

### 2.5. Material Characterization

PEDOT:PSS films and Ru(III) PEDOT:PSS complex films were used for material characterization. SEM images were obtained by a Hitachi SU5000 (Tokyo, Japan) at an accelerating voltage of 5 kV equipped with an EDS detector (Oxford, UK). TEM images were obtained by a Talos F200X (Waltham, MA, USA) at an accelerating voltage of 300 kV equipped with an EDS detector. The XRD test was conducted by an Empyrean at room temperature in the 2θ range of 20°~70° with a step size of 4°/min. The FTIR test was conducted by a Fourier transform infrared attenuated total reflection absorption spectrometer (FTIR-ATR, Nicolet iS50, Waltham, MA, USA). Raman spectroscopy analysis (Renishaw inVia-Reflex, London, UK) was carried out with a confocal Raman microscope in the spectral range 50~1200 cm^−1^, consisting of a 532 nm HeNe laser excitation source. XPS was conducted on a Thermo Scientific Nexsa (Waltham, MA, USA) spectrometer equipped with a monochromatic Al Kα X-ray source, and high-resolution spectra were charge-corrected to a peak C 1 s binding energy of 284.8 eV.

### 2.6. Electrode and Supercapacitor Characterization

The optical transmittances of the samples were measured by a UV−VIS spectrophotometer (Shimadzu UV-2600, Kyoto, Japan). In all cases, air was used as the reference. The electrochemical properties were measured with an electrochemical workstation (CHI660, Shanghai, China). A three-electrode configuration was used for measuring the electrochemical properties of the electrodes, consisting of PEDOT:PSS or Ru(III) PEDOT:PSS complex electrode as the working electrode, platinum plate as the counter electrode, Ag/AgCl as the reference electrode, and 1 M Na_2_SO_4_ aqueous solution as the electrolyte. The electrochemical performance of the supercapacitor devices was measured using a two-electrode configuration. The areal capacitance was derived from the CV curve using the following Equation (1):

(1) $$C_{A}=\frac{\int_{0}^{2V_{O}/v}\left|i\right|dt}{2V_{O}}$$

where $C_{A}$ is the areal capacitance (mF cm^−2^),$\;V_{O}$ is the potential window (V), v is the scan rate (mV s^−1^), i is the current density (mA cm^−2^), and t is the time (s). The energy density and power density are obtained by the following Equations (2) and (3), respectively:

(2) $$E=\frac{1000}{2\cdot 3600}C_{A}V_{O}^{2}$$

(3) $$P=3600\frac{E}{\Delta t}$$

where E is the areal energy density (μWh cm^−2^), P is the areal power density (μW cm^−2^), and Δt is the discharge time (s).

To evaluate the stretchability of the supercapacitor, mechanical deformation was carried out on an in-house motorized linear stage. Two platforms are mounted on the lead screw and bearings, with the lead screw converting the rotational motion of the motor into linear motion and the bearings guiding the movement. One platform is fixed, while the other platform can move linearly along the screw under the drive of a stepper motor. Two ends of the supercapacitor were fixed on the platforms, and then uniform strain/release cycles were applied to the sample with a speed of 1 mm s^−1^. The electrochemical measurement was conducted when the device was under specified strain.

## 3. Results

**Figure 1:**
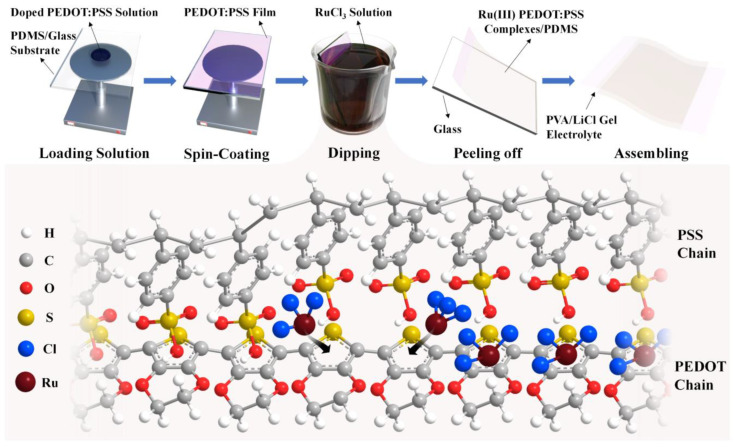
Schematic diagram of the fabrication process flow of the Ru(III) PEDOT:PSS complex stretchable and transparent supercapacitor. The schematic diagram below illustrates the complexation of Ru with the thiophene rings during the dipping process.

Figure 1 illustrates the methodological approach for fabricating stretchable and transparent supercapacitors utilizing Ru(III) PEDOT:PSS complexes. The process was initiated with pouring poly(dimethylsiloxane) (PDMS) onto a glass plate due to its favorable stretchability and transparency. After curing, the PDMS/glass substrate was spin-coated with doped PEDOT:PSS solution. Subsequently, immersing the coated substrate in RuCl_3_ solution for 40 s induced the formation of Ru(III) PEDOT:PSS complexes. The final assembly involved detaching the PDMS film and aligning two such films with a LiCl/PVA gel electrolyte in between, thereby constructing the supercapacitor.

### 3.1. Characterization of Ru(III) PEDOT:PSS Complexes

**Figure 2:**
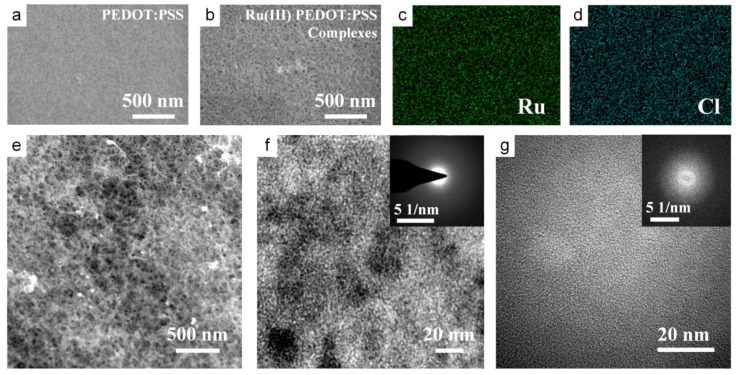
The morphological and structural information for the materials. SEM images of the (**a**) PEDOT:PSS film and (**b**) Ru(III) PEDOT:PSS complex film. EDS mappings of (**b**): (**c**) Ru and (**d**) Cl. (**e**) TEM image; (**f**) HRTEM and corresponding SAED image; (**g**) HRTEM and corresponding FFT image of Ru(III) PEDOT:PSS complexes.

To analyze the morphological and compositional information for the materials, scanning electron microscopy (SEM) was applied to observe the difference of the undoped PEDOT:PSS film before and after RuCl_3_ solution immersion, as shown in Figure 2a,b. The smooth surface of PEDOT:PSS film became rough after dipping in RuCl_3_ solution due to the formation of uniformly distributed pores with a diameter of less than 30 nm through the reaction. As the dipping time increased, the pores became more in number and larger (Figure S1). The porous structure of the material contributed significantly to its electrochemical performance by providing an enlarged specific surface area. The energy dispersive X-ray spectroscopy (EDS) mapping images of Ru(III) PEDOT:PSS complex films (Figure 2c,d) revealed that Ru and Cl had a uniform distribution in the film. The morphologies and structures of the as-synthesized film were further investigated by transmission electron microscope (TEM), as shown in Figure 2e–g and Figure S2. The selected area electron diffraction (SAED) image and the fast Fourier transformation (FFT) image indicate that the reaction products were amorphous, corresponding to the X-ray diffraction (XRD) pattern (Figure S3).

**Figure 3:**
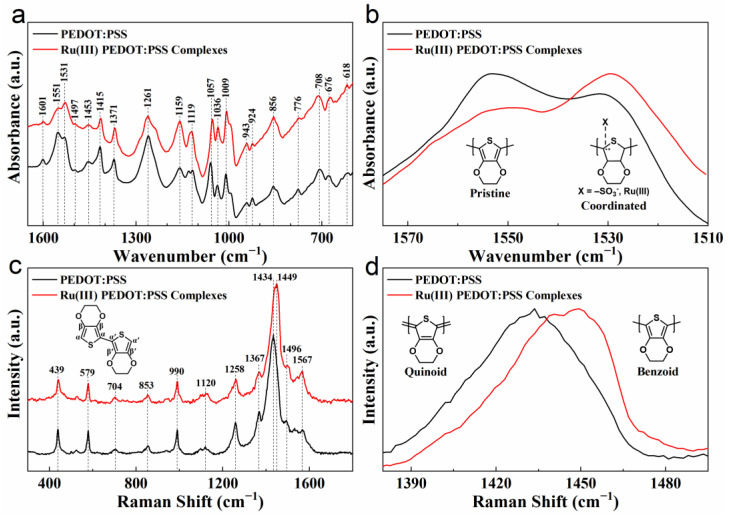
Structural characteristics of PEDOT:PSS and Ru(III) PEDOT:PSS complexes. (**a**) FTIR spectra of PEDOT:PSS and Ru(III) PEDOT:PSS complexes. (**b**) FTIR spectra related to the thiophene ring on PEDOT:PSS. (**c**) Raman spectra of PEDOT:PSS and Ru(III) PEDOT:PSS complexes. (**d**) Raman spectra related to the thiophene ring on PEDOT:PSS.

The chemical structures and functional groups of undoped PEDOT:PSS and Ru(III) PEDOT:PSS complexes were analyzed using Fourier transform infrared spectroscopy (FTIR), as illustrated in Figure 3a. Table S1 provides detailed peak assignments in line with the peaks observed in the spectrum. The FTIR spectral analysis confirmed that all regions possessed similar chemical compositions, with the most distinctive bands for PEDOT and PSS chains clearly identifiable. This implies that the incorporation of Ru did not change the covalent bonding of PEDOT:PSS. Notably, the intensities of the C=C stretching doublet (1551 and 1531 cm^−1^) changed after the addition of Ru, as depicted in Figure 3b, due to a shift in the ratio of pristine thiophene (1551 cm^−1^, decrease) to coordinated thiophene (1531 cm^−1^, increase). This change indicates that Ru complexed with the thiophene ring in PEDOT:PSS, wherein the thiophene ring served as an electron donor, while the Ru atom acted as an electron acceptor.

These materials were also examined using Raman spectroscopy in the range from 300 to 1800 cm^−1^, and the spectra are shown in Figure 3c. Table S2 provides detailed peak assignments in line with the peaks observed in the spectrum. The position of most of the peaks in the Ru(III) PEDOT:PSS complexes was consistent with those in the PEDOT:PSS, suggesting that the addition of Ru did not alter the main structure of PEDOT:PSS but only complexed with the thiophene ring in the PEDOT chain. However, it is worth noting that the peak at 1434 cm^−1^ shifted to 1449 cm^−1^ after the addition of Ru, as shown in Figure 3d. This indicates that the quinoid structure in PEDOT was transformed into a benzoid structure, and this structural change usually leads to the transformation of PEDOT chains from linear to core [29,30]. This may explain why the morphology of PEDOT:PSS became porous after the addition of Ru.

**Figure 4:**
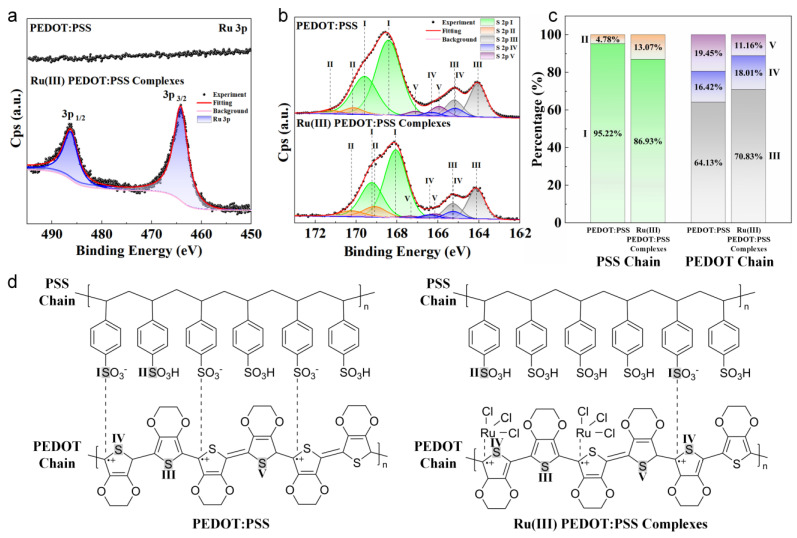
XPS spectra analysis and molecular structures of PEDOT:PSS and Ru(III) PEDOT:PSS complex films. (**a**) Ru 3p spectra. (**b**) S 2p spectra. (**c**) Composition ratio derived from (**b**). (**d**) Proposed molecular structures before and after PEDOT:PSS interacted with RuCl_3_. The labels ‘I’ to ‘V’ in (**b**) to (**d**) represent different states of sulfur atoms.

The chemical environment of undoped PEDOT:PSS and Ru(III) PEDOT:PSS complex films was investigated using X-ray photoelectron spectroscopy (XPS). The comprehensive XPS survey (Figure S4) detected the presence of Ru, Cl, C, O, and S elements on the surface of the Ru(III) PEDOT:PSS complex film, in contrast to the PEDOT:PSS film alone, which only exhibited C, O, and S elements. Figure 4a and Figure S5 display the XPS spectra for the Ru 3p and Cl 2p regions in PEDOT:PSS and Ru(III) PEDOT:PSS complex films, respectively. These figures clearly show the absence of Ru and Cl peaks in the PEDOT:PSS film, whereas distinct doublet peaks for Ru (464.16 eV) and Cl (198.12 eV) were observed in the Ru(III) PEDOT:PSS complex film, signifying the interaction of Ru and Cl atoms onto the PEDOT:PSS polymer matrix. Furthermore, the two peaks with binding energies of 464.16 and 486.27 eV (Figure 4a), for Ru 3p _3/2_ and Ru 3p _1/2_, confirmed a +3 oxidation state for Ru [31].

Regarding the impact of the incorporation of Ru(III) upon the polymer matrix, the S 2p spectra detailed in Figure 4b reveal two primary peaks between 166.5–172 eV and 163–166.5 eV, corresponding to the sulfur atoms within the PSS and PEDOT chains, respectively. Within the PSS chains, two distinct doublets were observed: the lower binding energy doublet represents sulfur in the –SO_3_^−^ form (labeled ‘I’), while the doublet with higher binding energy pertains to sulfur in the –SO_3_H form (labeled ‘II’) [32]. The intensity of the photoelectron peaks in the XPS spectra was proportional to the concentration of each element state, allowing for the quantitative determination of elemental composition. The proportions of different sulfur atom states in the PSS chain or PEDOT chain were calculated and are plotted in Figure 4c. A comparison with the standard PEDOT:PSS shows an increase in the proportion of –SO_3_H (labeled ‘II’) in the Ru(III) PEDOT:PSS complexes, from 4.78% to 13.07%, as depicted in Figure 4c. This suggests that Ru(III) complexation leads to substituting some –SO_3_^−^ within the PSS chains that interacted with thiophene rings within the PEDOT chains by Ru(III), resulting in the formation of –SO_3_H. On the other hand, in the PEDOT chains, the S 2p spectrum was comprised of three doublets, aligning with previous studies [32]. The doublet around 164.1 eV (labeled ‘III’) is associated with sulfur in the neutral (pristine) thiophene rings of the benzoid (aromatic) structure [33,34]. Another doublet at approximately 165.2 eV (labeled ‘IV’) corresponded to positively polarized or partially charged sulfur species, emerging from charge extraction from thiophene units by either –SO_3_^−^ or the Ru(III) dopant [35,36,37,38]. The third doublet, around 166.0 eV (labeled ‘V’), is attributed to sulfur in a more oxidized quinoid structure [33,34]. Figure 4c indicates a decrease in the quinoid structures (labeled ‘V’) within the PEDOT chains of the complexes, shifting towards positively polarized or partially charged (labeled ‘IV’) and benzoid structures (labeled ‘III’), corroborating the insights gained from Raman spectroscopy analyses. From the analysis conducted, we proposed a molecular structure for the Ru(III) PEDOT:PSS complexes, as depicted in Figure 4d (the highlighted sulfur atoms with labels “I” to “V” correspond to the different states of sulfur atoms in Figure 4b,c). When compared to the PEDOT:PSS structure [39,40], three significant modifications can be observed in the Ru(III) PEDOT:PSS complexes: (1) Ru(III) formed a complex with the thiophene ring; (2) Ru(III) replaced part of the –SO_3_^−^ groups that were initially interacting with the thiophene ring, resulting in the formation of –SO_3_H; and (3) the configuration of the PEDOT chains transitioned from quinoid to benzoid structures.

### 3.2. Properties of Ru(III) PEDOT:PSS Complex Electrodes

**Figure 5:**
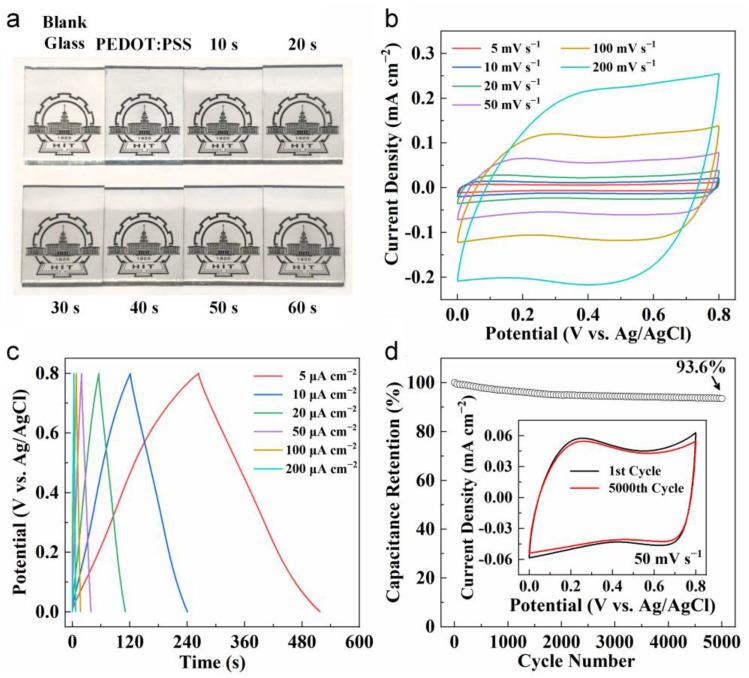
Optical and electrochemical performance of Ru(III) PEDOT:PSS complex electrodes. (**a**) Digital photographs of blank glass, PEDOT:PSS electrode, and Ru(III) PEDOT:PSS complex electrodes with different dipping times. (**b**) Cyclic voltammetry (CV) curves (scan rate: 5–200 mV s^−1^) and (**c**) galvanostatic charge/discharge (GCD) curves (current density: 5–200 μA cm^−2^) of the Ru(III) PEDOT:PSS complex electrode with a dipping time of 40 s. (**d**) Capacitance retention of the Ru(III) PEDOT:PSS complex electrode under 5000 repeated CV cycling test. The inset shows the CV curves of the initial and final cycle.

To evaluate the optical and electrochemical performance of PEDOT:PSS and Ru(III) PEDOT:PSS complex electrodes, doped PEDOT:PSS was spin-coated on the glass substrate, followed by dipping in the RuCl_3_ solution for different time periods (10–60 s). We used 6 wt% EG and 2 wt% Triton-X 100 to dope the PEDOT:PSS solution [30,41]. Digital photographs of blank glass, PEDOT:PSS electrode, and Ru(III) PEDOT:PSS complex electrodes with different dipping times are shown in Figure 5a. The PEDOT:PSS electrode was light blue, while being dipped in RuCl_3_ solution resulted in it becoming a faint reddish-brown hue. As the dipping time increased, the color gradually deepened while maintaining relatively high transparency. Transmittance spectra for blank glass and these electrodes are presented in Figure S6, demonstrating high transparency (more than 80%) across all electrodes after introducing Ru(III).

The electrochemical properties of the PEDOT:PSS electrode and various Ru(III) PEDOT:PSS complex electrodes were firstly evaluated by a standard three-electrode configuration at room temperature, containing a working electrode, a platinum plate counter electrode, a Ag/AgCl reference electrode, and a 1 M Na_2_SO_4_ electrolyte. Taking Ru(III) PEDOT:PSS complexes with a dipping time of 40 s as an example, Figure 5b shows the CV curves at a scan rate from 5 to 200 mV s^−1^. The nearly rectangular shaped CV curves exhibited the excellent charge storage properties and swift response of the Ru(III) PEDOT:PSS complex electrode, maintaining this shape even at a high scan rate of 200 mV s^−1^. In addition to the CV analysis, the GCD curves at various current densities ranging from 5 to 200 μA cm^−2^ displayed almost symmetrical characters without apparent IR drop, presenting a near triangle shape (Figure 5c). These results indicate outstanding electrochemical capacitive characteristics of the electrodes, stemming from the reversible Faradaic reactions between the Ru(III) PEDOT:PSS complexes and electrolyte.

In addition, stability during the charge/discharge cycling process is crucial for the practical utilization of supercapacitors. Therefore, a charge/discharge cycling experiment was conducted to assess the electrochemical stability of the Ru(III) PEDOT:PSS complex electrode. Compared to its initial state, the electrode retained 93.6% capacitance after undergoing 5000 cycles of repetitive CV cycling at 50 mV s^−1^, as demonstrated in Figure 5d. The electrode showed only a slight performance decay in the CV curve, as shown in the inset of Figure 5d, demonstrating its excellent electrochemical cycling stability and long cycle life. Moreover, the electrochemical stability of Ru(III) PEDOT:PSS complexes is superior to PEDOT:PSS (remaining 87% after 5000 charge-discharge cycles) [28]. On the one hand, the performance decay of PEDOT:PSS mainly came from the expansion and contraction during the charge/discharge process (doping–dedoping) [42,43]. Compared to the PEDOT:PSS, the porous morphology of the Ru(III) PEDOT:PSS complex film would buffer this volumetric change during cycling, therefore improving the stability. On the other hand, the electrochemical stability of Ru compounds is superior to PEDOT:PSS [28,44]. Therefore, the incorporation of Ru(III) may help enhance the electrochemical stability.

**Figure 6:**
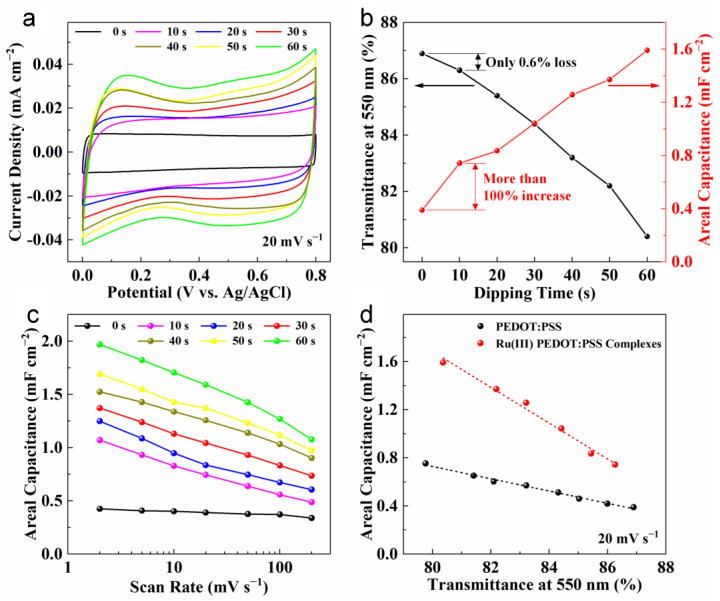
Comparison of optical and electrochemical performance between PEDOT:PSS and Ru(III) PEDOT:PSS complex electrodes. (**a**) CV curves of the PEDOT:PSS electrode and Ru(III) PEDOT:PSS complex electrodes with different dipping times (scan rate = 20 mV s^−1^). (**b**) Transmittance at 550 nm (using air as the reference) and areal capacitance versus Ru(III) PEDOT:PSS complex dipping time relationships. (**c**) The rate performances of different electrodes in terms of areal capacitance, which were obtained by the CV curves. (**d**) The plot of areal capacitance as a function of the electrode transmittance at 550 nm (scan rate = 20 mV s^−1^).

To compare the capacitance properties between PEDOT:PSS and Ru(III) PEDOT:PSS complex electrodes, the relationship between the dipping time and the electrochemical performance was examined. Figure 6a presents CV curves of various electrodes with a scan rate of 20 mV s^−1^ (0 s represents the PEDOT:PSS electrode). The CV curve of the PEDOT:PSS sample exhibited an ideal rectangular shape, indicating an optimal capacitive response. As the dipping time increased, the CV encircled area significantly expanded, likely attributed to the pseudocapacitive Ru(III) in the complexes. The current response upon reversal of the scan direction remained swift, even if the dipping time was 60 s, suggesting a short time constant for charge and discharge. The energy storage mechanism may have been rapid and reversible electron transfer on the surface of Ru(III) PEDOT:PSS complexes, where a redox reaction occurred between the III and IV oxidation states of Ru ions. Despite limited research on Ru(III) compounds as capacitive materials, their pseudocapacitance was recognized as early as 1995. Zheng et al. found that powder precipitated from a RuCl_3_ solution and NaOH solution mixture at room temperature exhibited a capacitance of 527 F g^−1^ [45]. This was 73.2% of the capacitance of hydrous RuO_2_ produced by sintering the precipitated powder at 150 °C.

To examine the correlation between the optical and electrochemical properties of PEDOT:PSS and Ru(III) PEDOT:PSS complex electrodes, we analyzed their transparency at 550 nm and areal capacitance, as shown in Figure 6b. As the dipping time increased, there was a consistent decrease in optical transmittance alongside an increase in areal capacitance. Transparency at 550 nm diminished from 91.1% for bare glass to 86.9% for the PEDOT:PSS electrode, and further to between 86.3% and 80.4% for the Ru(III) PEDOT:PSS complex electrode over dipping times from 10 s to 60 s. Concurrently, areal capacitance rose from 0.39 mF cm^−2^ for the PEDOT:PSS electrode to between 0.74 and 1.59 mF cm^−2^ for the complex electrode. Notably, a mere 10 s of dipping resulted in only a 0.6% decrease in optical transmittance at 550 nm, while areal capacitance increased by more than double, highlighting the significant impact of Ru(III) complexation on the electrochemical properties.

Figure 6c depicts the rate performances of PEDOT:PSS and different Ru(III) PEDOT:PSS complex electrodes. Throughout the entire testing range (2–200 mV s^−1^), the areal capacitances steadily enlarged with the increasing dipping time. The areal capacitance of the Ru(III) PEDOT:PSS complex electrode with a dipping time of 60 s achieved 1.97 mF cm^−2^ at a scan rate of 2 mV s^−1^, 4.6 times that of the PEDOT:PSS electrode. Even at a high scan rate of 200 mV s^−1^, the areal capacitance of the Ru(III) PEDOT:PSS complex electrode was retained at 1.08 mF cm^−2^, which was 3.2 times that of the PEDOT:PSS electrode, showing a suitable rate capacitance of 54.8%. Although the capacitance of the Ru(III) PEDOT:PSS complex electrode was much larger than that of the PEDOT:PSS electrode, the transparency slightly decreased. In order to compare the capacitance of these two materials under similar transparency, PEDOT:PSS electrodes with different thicknesses and transparency were prepared by adjusting the spin coating speed. The areal capacitance obtained from 20 mV s^−1^ CV curves is plotted as a function of transparency at 550 nm, as shown in Figure 6d. The Ru(III) PEDOT:PSS complex electrodes maintained 82 to 124% higher areal capacitance than the PEDOT:PSS electrodes at the similar transparency. This improvement also existed with the scan rate of 2 mV s^−1^ and 200 mV s^−1^ (Figure S7). These outstanding results are attributed to the synergistic advantages of Ru(III) and PEDOT:PSS in the Ru(III) PEDOT:PSS complexes, which mainly provided high pseudocapacitance and acted as a transparent current collector, respectively.

### 3.3. Properties of the Ru(III) PEDOT:PSS-Complex-Based Supercapacitor Device

**Figure 7:**
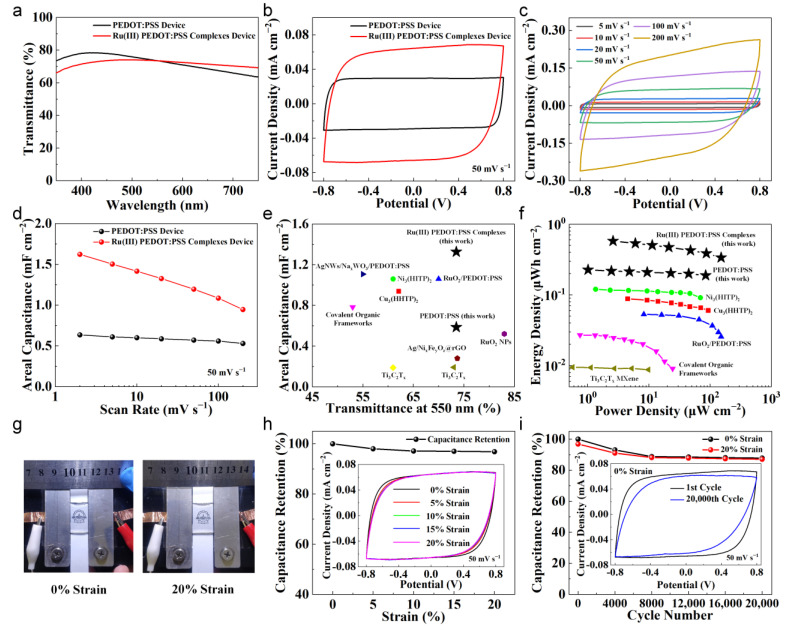
Characterizations of symmetric supercapacitor devices based on PEDOT:PSS or Ru(III) PEDOT:PSS complexes. (**a**) Transmittance spectra of these two devices (using air as the reference). (**b**) CV curves of these two devices (scan rate = 50 mV s^−1^). (**c**) CV curves of the device based on Ru(III) PEDOT:PSS complexes (scan rate: 5–200 mV s^−1^). (**d**) Areal capacitance comparison of these two devices at different scan rates (2–200 mV s^−1^). (**e**) Areal capacitance versus transmittance at 550 nm and comparison to other transparent supercapacitors [26,28,47,48,49,50,51,52,53]. (**f**) Ragone plots of these devices and comparison to other transparent supercapacitors [28,47,48,49,50]. (**g**) Digital photographs of the device based on Ru(III) PEDOT:PSS complexes with 0% and 20% strain. (**h**) Capacitance retention of the device based on Ru(III) PEDOT:PSS complexes under strain ranging from 0 to 20%. The inset shows the CV curves of the device with 0% to 20% strain (scan rate = 50 mV s^−1^). (**i**) Capacitance retention of the device based on Ru(III) PEDOT:PSS complexes during 20,000 stretching–relaxation cycles from 0% to 20% applied strain. The inset shows the CV curves of the device at 0% strain during the initial and final cycle (scan rate = 50 mV s^−1^).

To explore the applications of Ru(III) PEDOT:PSS complexes in stretchable and transparent energy storage devices, two solid-state symmetric supercapacitors based on PEDOT:PSS or Ru(III) PEDOT:PSS complexes were assembled, individually. Doped PEDOT:PSS was spin-coated on the PDMS substrate, then directly used or dipped in the RuCl_3_ solution for 40 s. We used 6 wt% EG, 2 wt% Triton-X 100, and 15 wt% Capstone FS-3100 to dope the PEDOT:PSS solution [46]. Two identical films were sandwiched with a PVA/LiCl gel electrolyte, forming a stretchable and transparent symmetric supercapacitor. The transmittance spectra of these devices in the visible region are illustrated in Figure 7a, demonstrating a comparable transparency at 550 nm (around 73.5%) in the two devices.

The electrochemical properties were evaluated by CV characterization with a two-electrode configuration at room temperature. The CV curves with 50 mV s^−1^ of these devices are plotted in Figure 7b, which indicates that the Ru(III) PEDOT:PSS-complex-based supercapacitor led to significantly higher capacitance, as seen in the noticeably enlarged encircled area under the CV curve. The CV curve exhibited an ideal rectangular shape, accompanied by a swift current response upon voltage reversal. Figure 7c shows the CV characterization of the Ru(III) PEDOT:PSS-complex-based supercapacitor at different scan rates from 2 mV s^−1^ to 200 mV s^−1^. The device showed well-shaped CV curves, even at a high scan rate of 200 mV s^−1^, demonstrating excellent energy capacitive performance and favorable rate capability. The Ru(III) PEDOT:PSS-complex-based device exhibited 1.62 mF cm^−2^ at 2 mV s^−1^ and decreased to 1.32 mF cm^−2^ and 0.95 mF cm^−2^ at 20 mV s^−1^ and 200 mV s^−1^, respectively (Figure 7d). Nevertheless, the areal capacitance of the Ru(III) PEDOT:PSS-complex-based device significantly exceeded that of the PEDOT:PSS-based device across all scan rate ranges, being 155%, 126%, and 79% higher at 2 mV s^−1^, 20 mV s^−1^, and 200 mV s^−1^, respectively.

As illustrated in Figure 7e, compared with other reported transparent supercapacitor devices [26,28,47,48,49,50,51,52,53], the as-fabricated Ru(III) PEDOT:PSS-complex-based supercapacitor showed considerable advantages in terms of areal capacitance and optical transmittance. Furthermore, the device yielded a remarkable maximum areal energy density of 0.58 μWh cm^−2^ at an areal power density of 2.60 μW cm^−2^, significantly exceeding those of previously reported transparent supercapacitors based on other materials, such as RuO_2_/PEDOT:PSS (0.053 μWh cm^−2^ at 8.27 μW cm^−2^) [28], Ni_3_(HITP)_2_ metal–organic frameworks (0.12 μWh cm^−2^ at 1.35 μW cm^−2^) [47], Cu_3_(HHTP)_2_ metal–organic frameworks (0.088 μWh cm^−2^ at 4.5 μW cm^−2^) [48], covalent organic frameworks (0.027 μWh cm^−2^ at 0.75 μW cm^−2^) [49], and Ti_3_C_2_T_x_ MXene (0.009 μWh cm^−2^ at 0.5682 μW cm^−2^) [50] (Figure 7f and Table S3). It firmly enhanced the potential of Ru(III) PEDOT:PSS-complex-based supercapacitors for application in next-generation portable transparent electronics.

Although exhibiting excellent electrochemical properties under optimal conditions, the stretchability of supercapacitors remains a crucial factor for wearable electronic devices. Consequently, we conducted additional investigations into the electrochemical behavior of the Ru(III) PEDOT:PSS-complex-based supercapacitor under mechanical deformation. Digital photographs of the device fixed on a motorized linear stage with 0% and 20% strain are shown in Figure 7g, wherein no evident damages can be observed in the device. The CV curves in Figure 7h show no noticeable change upon stretching from 0% to 20% using the linear stage with a speed of 1 mm s^−1^. The capacitance obtained from CV curves remained stable, exhibiting a mere 3.2% decrement. The slightly reduced capacitance may primarily stem from the degradation of contacts between the electrode and the gel electrolyte in the deformation process, leading to an expanded resistance of the whole supercapacitor.

Although the device maintained promising performance under deformation, it must be able to endure repeated stretching during practical application. To assess the stability of the Ru(III) PEDOT:PSS-complex-based supercapacitor in response to recurrent stretching, it underwent repeated stretching–relaxation cycles in which the strain ranged from 0% to 20%. CV measurements were conducted at 50 mV s^−1^ every 4000 cycles. Even after 20,000 stretching–relaxation cycles, the CV curve remained rectangular, showing no significant change (Figure 7i). The capacitance obtained from CV curves remained at 87.8% of its initial value. Most of the decrement in electrochemical performance occurred within the initial 4000 cycles (9.0% loss), and the capacitance remained relatively stable after 8000 cycles (11.8% loss). This behavior demonstrates that the contact between materials was maintained intact, and minimal film separation or impairment occurred during the cycling test, which is encouraging for implementing this device in practical applications. These results evidently demonstrate the high stretchability and stability of Ru(III) PEDOT:PSS-complex-based supercapacitors.

Table 1 comprehensively compares the transparent supercapacitor devices [26,28,47,48,49,50,51,52,53]. Compared with the devices reported, the Ru(III) PEDOT:PSS-complex-based supercapacitor in this work exhibited a much better comprehensive performance. Besides higher transmittance and areal capacitance, it simultaneously had better long-term electrochemical cycling stability and stretchability. Additionally, the Ru(III) PEDOT:PSS complexes discussed in this study hold tremendous potential for practical commercial applications. On the one hand, they offer the advantage of low cost. The synthesis process occurs at room temperature without the need of elevated temperature or pressure, which eliminates the requirement for complex equipment. Furthermore, the synthesis process is solution-processible, making it easily scalable for large-scale production, further reducing costs. Despite the relatively high cost of the raw material, RuCl_3_, its high utilization efficiency enables full participation in the reaction, resulting in minimal consumption. On the other hand, Ru(III) PEDOT:PSS complexes exhibit characteristics of efficient synthesis. The reaction was completed in just 40 s and can be further shortened by increasing the concentration of RuCl_3_ solution, which is several orders of magnitude lower than most reported works. These outstanding advantages indicate the promising future of Ru(III) PEDOT:PSS complexes for fabricating high-performance stretchable and transparent supercapacitors to power wearable electronics.

## 4. Conclusions

In conclusion, Ru(III) PEDOT:PSS complexes were readily synthesized by dipping PEDOT:PSS films in RuCl_3_ solution for no longer than one minute to construct stretchable and transparent supercapacitors. This simple and efficient synthesis method leverages the advantages of Ru(III) and PEDOT:PSS, which primarily contribute high pseudocapacitance and serve as a stretchable transparent current collector, respectively. The supercapacitor based on Ru(III) PEDOT:PSS complexes exhibited outstanding electrochemical performance. It achieved a capacitance of 1.62 mF cm^−2^ at a transmittance of 73.5%, which was 2.55 times that of the PEDOT:PSS-based supercapacitor with comparable transparency. Additionally, the device demonstrated an impressive maximum energy density of 0.58 μWh cm^−2^ at a power density of 2.60 μW cm^−2^. After 20,000 cycles of repetitive stretching from 0% to 20% strain, it retained 87.8% of its initial capacitance. This work demonstrates the potential of Ru(III) PEDOT:PSS complexes, with their simple synthesis and high performance, as a supercapacitor material in portable and wearable electronic devices.

## Figures and Tables

**Table 1:** Comprehensive comparison of different transparent supercapacitor devices.

Electrode Material	Transmittance at 550 nm	Areal Capacitance (mF cm^−2^)	Charge/Discharge Cycle Retention (Cycles)	Mechanical Performance	Synthesis Method	Synthesis Condition	Ref.
Ru(III) PEDOT:PSS complexes	73.5% vs. air	1.62	93.6% (5000) (electrode)	Stretchable, 20% strain	Dipping method	Ambient conditions 40 s	This work
AgNWs/NaxWO_3_/ PEDOT:PSS	55% vs. air	1.107	80% (2000)	Just flexible	Hydrothermal method	180 °C 20 h	[26]
RuO_2_/PEDOT:PSS	78% vs. PET (≈70% vs. air)	1.06	93% (6000)	Just flexible	Hydrothermal method	180 °C 6 h	[28]
Ni_3_(HITP)_2_	61% vs. air	1.06	87.32% (4000)	Just flexible	Modified air/liquid interfacial method	60 °C 30 min	[47]
Cu_3_(HHTP)_2_	62.1% vs. air	0.939	85% (3000)	Just flexible	Layer-by-layer assembly method	Ambient conditions 75 s	[48]
Covalent organic frameworks	53% vs. air	0.784	100% (3400)	Just flexible	Nucleophilic aromatic substitution reactions	120 °C 72 h	[49]
Ti_3_C_2_T_x_	61% vs. glass (≈55% vs. air)	0.189	90% (10,000)	Just rigid	Modified minimally intensive layer delamination method	48 h	[50]
RuO_2_ NPs	92.3% vs. ITO/glass (≈83% vs. air)	0.52	93.5% (5000)	Just rigid	Phase-transfer and precipitation technique	Room temperature 15 h, then annealing at 200 °C	[51]
Ag/Ni_x_Fe_y_O_z_@rGO	73.7% vs. air	0.282	90.4% (1000)	Just flexible	Microwave-assisted method	160 °C 30 min	[52]
Ti_3_C_2_T_x_	73%	0.192	85% (10,000) (electrode)	Just flexible	Pressure-less sintering method	30 MPa 1350 °C 1 h	[53]

## Data Availability

The data presented in this study are available on request from the corresponding author.

## Supplementary Materials

The following supporting information can be downloaded at https://www.mdpi.com/article/10.3390/nano14100866/s1. Figure S1: SEM images of the PEDOT:PSS film and Ru(III) PEDOT:PSS complex films with different dipping times. (a,b) PEDOT:PSS film. (c,d) Dipping 10 s. (e,f) Dipping 20 s. (g,h) Dipping 30 s. (i,j) Dipping 60 s. Figure S2: (a) TEM and (b,c) HRTEM images of Ru(III) PEDOT:PSS complex film. (d) HAADF image of Ru(III) PEDOT:PSS complexes and corresponding element mappings of (e) Ru, (f) Cl, (g) C, (h) O, and (i) S elements. Figure S3: XRD pattern of Ru(III) PEDOT:PSS complex film. Figure S4: XPS survey spectra of PEDOT:PSS and Ru(III) PEDOT:PSS complex films. Figure S5: XPS Cl 2p spectra of PEDOT:PSS and Ru(III) PEDOT:PSS complex films. Figure S6: Transmittance spectra (350–750 nm) of the blank glass, PEDOT:PSS electrode, and Ru(III) PEDOT:PSS complex electrodes with different dipping times from 10 to 60 s, using air as the reference. Figure S7: The plot of areal capacitance as a function of the electrode transmittance at 550 nm (using air as the reference). (a) Scan rate = 2 mV s^−1^. (b) Scan rate = 200 mV s^−1^. Table S1: FTIR peak assignment corresponding to the PEDOT:PSS and Ru(III) PEDOT:PSS complexes. Table S2: Raman peak assignment corresponding to the PEDOT:PSS and Ru(III) PEDOT:PSS complexes. Table S3: Energy density and power density of transparent supercapacitor devices in Figure 7f. References [54,55,56,57,58,59,60] are cited in the Supplementary Materials.
