# Supplementary material for: Simple and Efficient Synthesis of Ruthenium(III) PEDOT:PSS Complexes for High-Performance Stretchable and Transparent Supercapacitors
Source: Nanomaterials (Basel). 2024 May 16;14(10):866. doi: 10.3390/nano14100866 (PMC11124221; doi:10.3390/nano14100866)
Supplement: Supplementary file 1 [file nanomaterials-14-00866-s001.zip › nanomaterials-2994005-supplementary.pdf]

Electronic Supplementary Materials

Simple and Efficient Synthesis of Ruthenium(III) PEDOT:PSS Complexes for High-performance Stretchable and Transparent Supercapacitors

Guiming Liu [1], Zhao Huang [1], Jiujie Xu [1], Bowen Zhang [2,*], Tiesong Lin [1,*], Peng He [1,*]

1 State Key Laboratory of Precision Welding & Joining of Materials and Structures, Harbin Institute of
Technology, Harbin 150001, China; liu.guiming@outlook.com (G.L.);
huangzhaohit@outlook.com (Z.H.); 20b909121@stu.hit.edu.cn (J.X.)
2 School of Electrical Engineering, Tiangong University, Tianjin 300350, China;
bowenzhang@tju.edu.cn
* Correspondence: hitjoining@hit.edu.cn (T.L.); hithepeng@hit.edu.cn (P.H.)

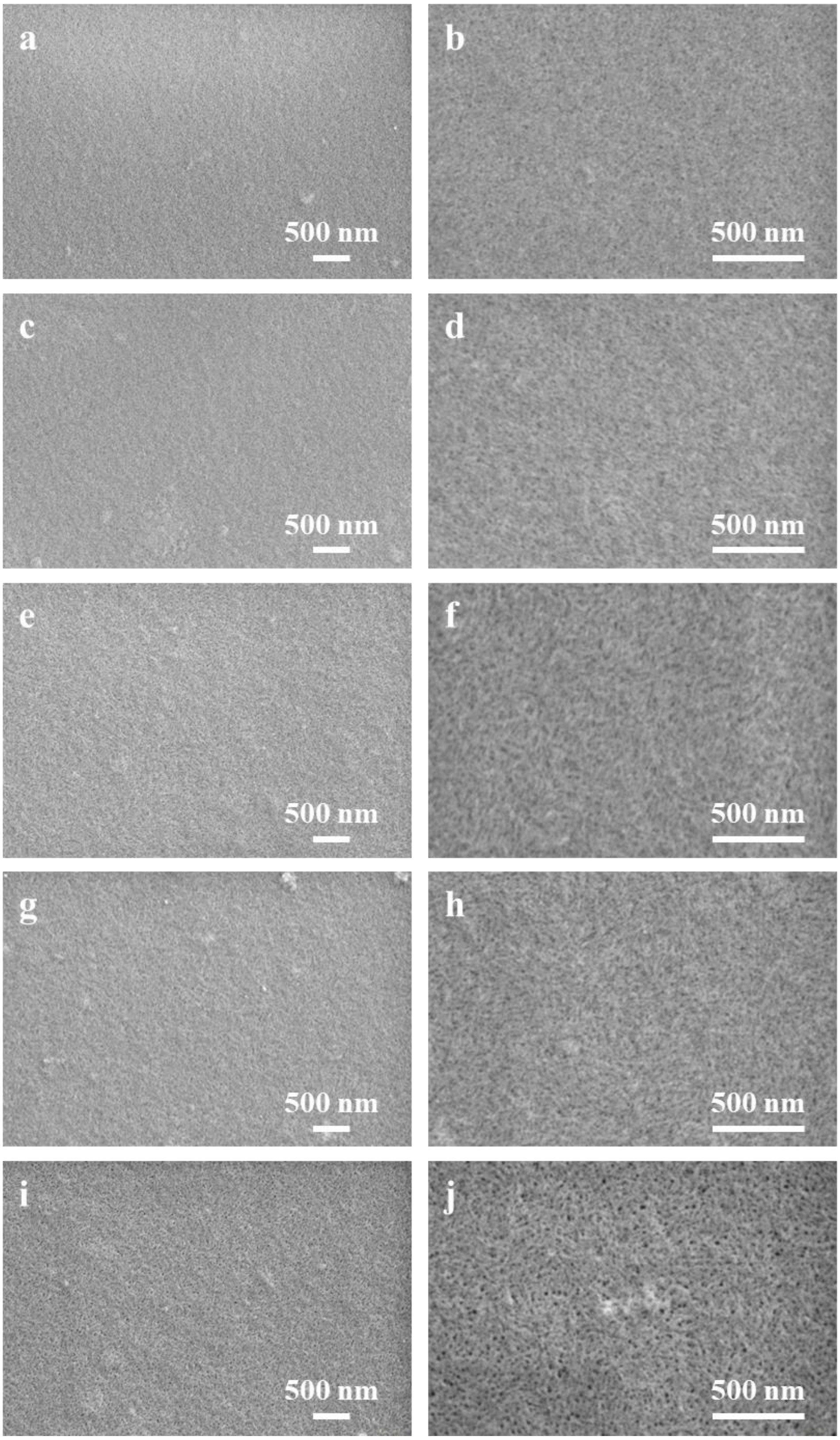

Figure S1. SEM images of the PEDOT:PSS film and Ru(III) PEDOT:PSS complexes films with different dipping time. (a) and (b) PEDOT:PSS film. (c) and (d) Dipping 10 s. (e) and (f) Dipping 20 s. (g) and (h) Dipping 30 s. (i) and (j) Dipping 60 s.

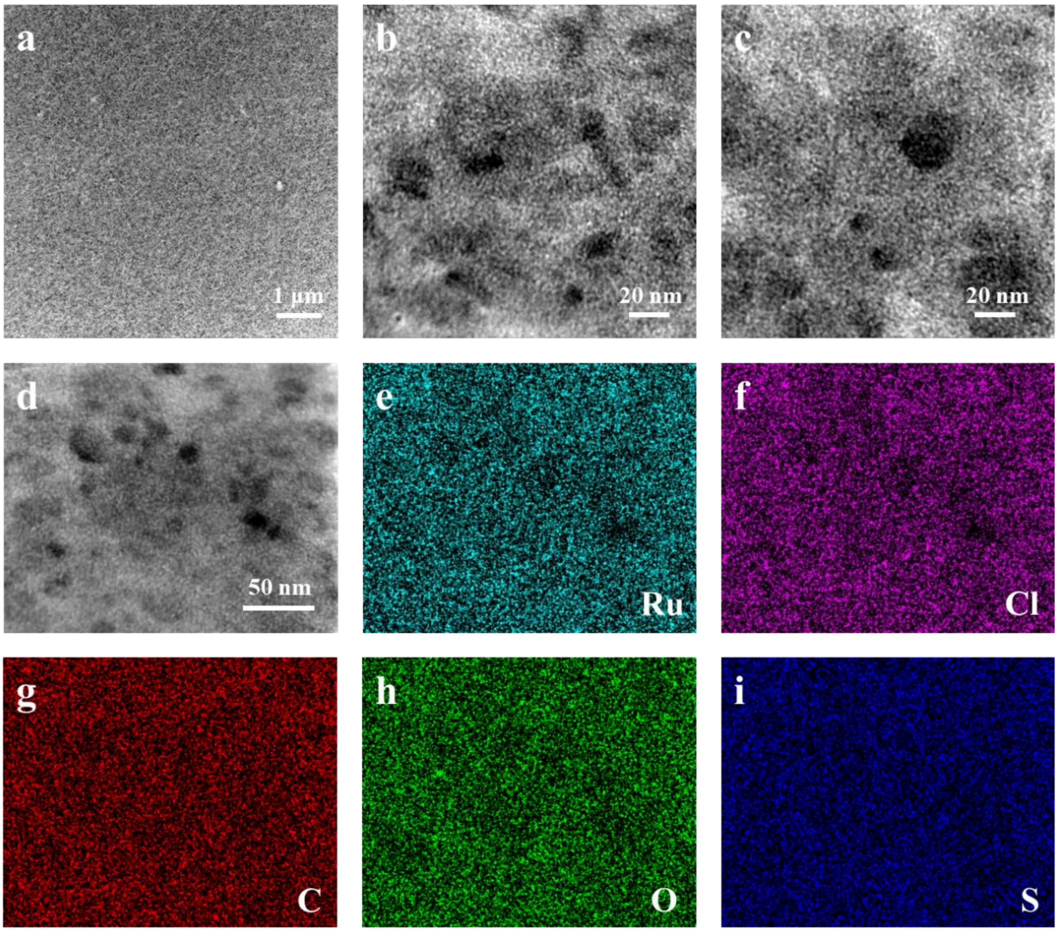

Figure S2. (a) TEM and (b, c) HRTEM images of Ru(III) PEDOT:PSS complexes film. (d) HAADF image of Ru(III) PEDOT:PSS complexes and corresponding element mappings of (e) Ru, (f) Cl, (g) C, (h) O and (i) S elements.

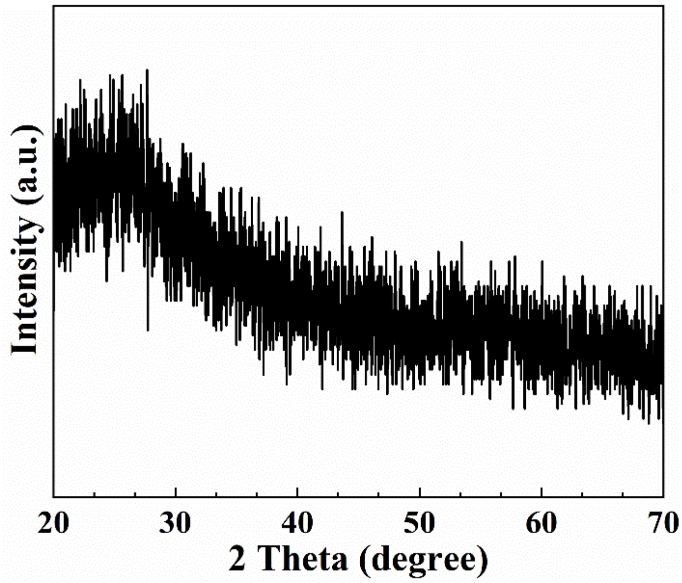

Figure S3. XRD pattern of Ru(III) PEDOT:PSS complexes film.

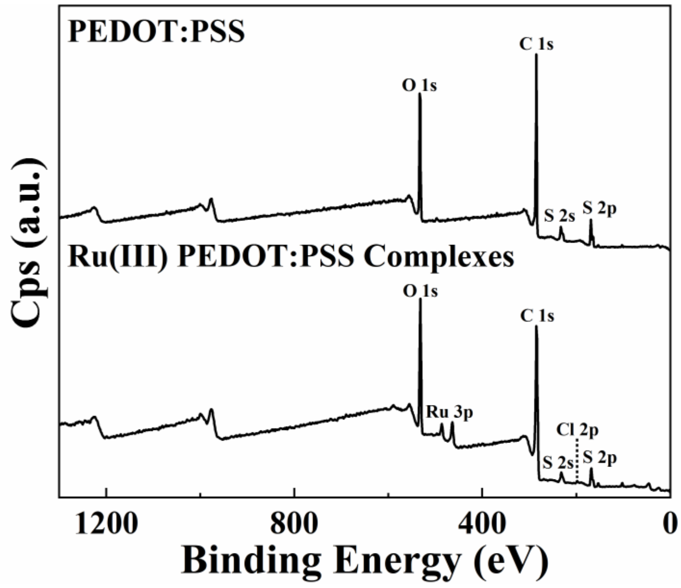

Figure S4. XPS survey spectra of PEDOT:PSS and Ru(III) PEDOT:PSS complexes films.

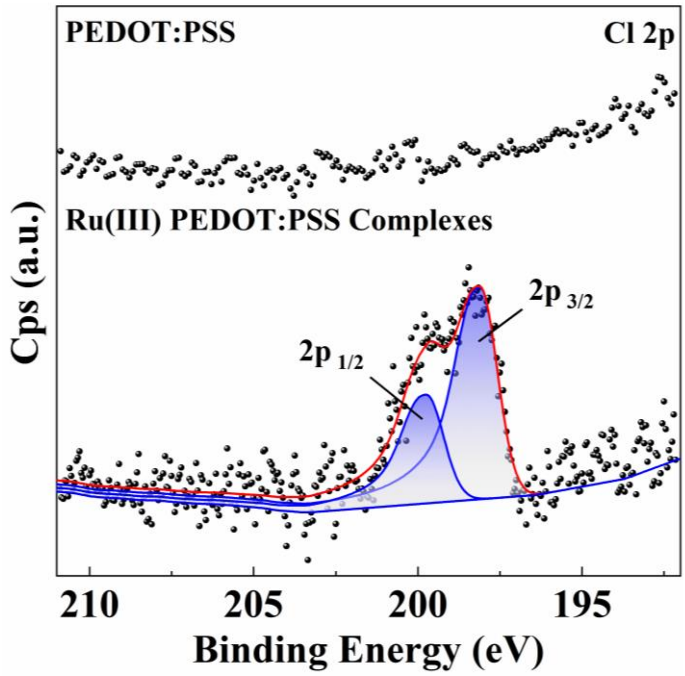

Figure S5. XPS Cl 2p spectra of PEDOT:PSS and Ru(III) PEDOT:PSS complexes films.

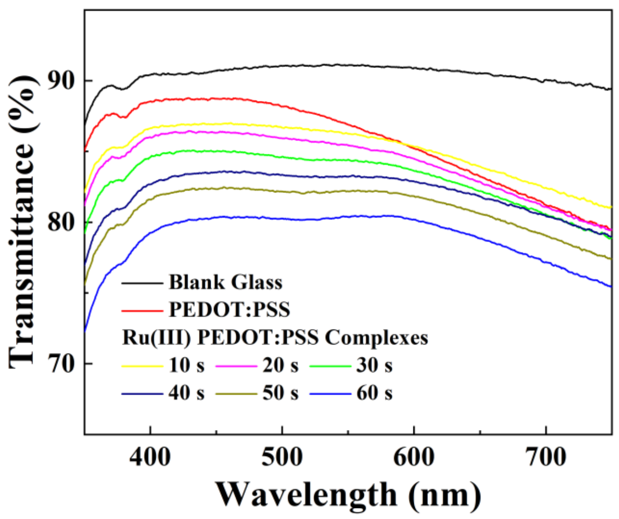

Figure S6. Transmittance spectra (350-750 nm) of the blank glass, PEDOT:PSS electrode and Ru(III) PEDOT:PSS complexes electrodes with different dipping time from 10 to 60 s, using air as the reference.

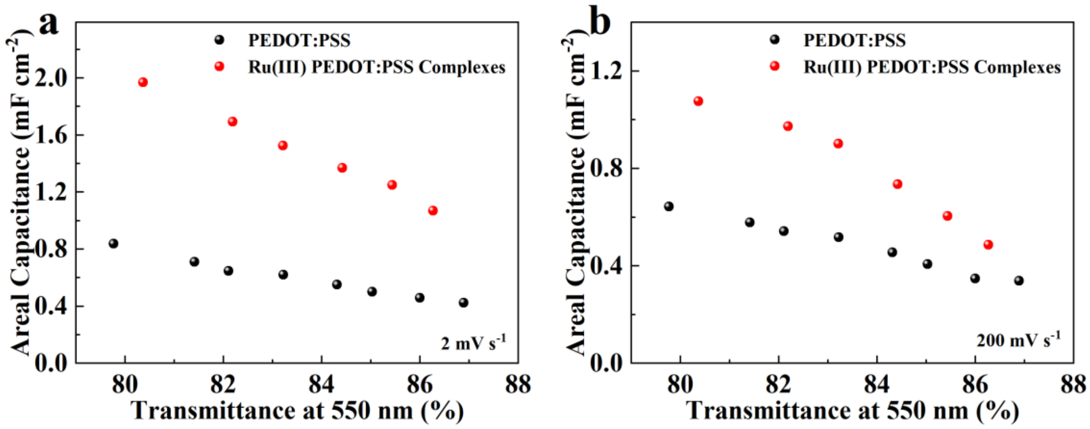

Figure S7. The plot of areal capacitance as a function of the electrode transmittance at 550 nm (using air as the reference). (a) Scan rate = 2 mV s^{-1}. (b) Scan rate = 200 mV s^{-1}.

Table S1. FTIR peak assignment corresponding to the PEDOT:PSS and Ru(III) PEDOT:PSS complexes .

Peaks [cm^{-1}]	Corresponding bonds	Ref.
1551 and 1531	Asymmetric C=C stretching within the thiophene ring, PEDOT	[1, 2]
1415	Symmetric C=C stretching, PEDOT	[1]
1371	C–C stretching within the thiophene ring, PEDOT	[1, 2]
1261 and 1130	Symmetric C–O–C stretching, PEDOT	[1-3]
1057	Asymmetric C–O–C stretching, PEDOT	[2]
924	Deformation of the ethylenedioxy group in the molecule, PEDOT	[3]
943 and 856	C–S stretching, PEDOT	[2, 3]
1601, 1497, 1453	Symmetric C=C stretching, PSS	[2-4]
1159 and 1119	Asymmetric SO_3 stretching, PSS	[1, 2, 4]
1036 and 1009	Symmetric SO_3 stretching, PSS	[2, 4]
776, 708, 676	C–H out-of-plane deformation, PSS	[4]
618	Ring in-plane deformation, PSS	[4]

Table S2. Raman peak assignment corresponding to the PEDOT:PSS and Ru(III) PEDOT:PSS complexes.

Peaks [cm^{-1}]	Corresponding bonds	Ref.
439		[5-7]
579 and 990	oxyethylene ring deformation, PEDOT	[5-7]
704	symmetric C–S–C deformation, PEDOT	[5-7]
853	C–H bending, PEDOT	[7]
1120	C–O–C deformation, PEDOT	[5-7]
1258	C_{α}–$C_{\alpha'}$ inter-ring stretching, PEDOT	[5-7]
1367	C_{β}–C_{β} stretching, PEDOT	[5-7]
1434 and 1449	C_{α}=C_{β} symmetrical stretching, PEDOT	[5-7]
1496	C_{α}=C_{β} asymmetrical stretching, PEDOT	[5, 6]
1567	C_{α}=C_{β} antisymmetrical stretching, PEDOT	[6, 7]

Table S3. Energy density and power density of transparent supercapacitor devices in Figure 7f.

Materials	Power Density [μW cm^{-2}]	Energy Density [μWh cm^{-2}]	Ref.
Ru(III) PEDOT:PSS complexes	2.60	0.58	This work
	6.01	0.53	
	11.33	0.50	
	21.21	0.47	
	47.82	0.43	
	86.83	0.39	
	151.35	0.34	
PEDOT:PSS	1.02	0.226	This work
	2.44	0.217	
	4.80	0.213	
	9.38	0.208	
	22.81	0.203	
	44.72	0.199	
	84.65	0.188	
RuO_2/PEDOT:PSS	8.27	0.053	[8]
	16.14	0.052	
	30.88	0.051	
	66.01	0.045	
	108.68	0.037	
	136.66	0.030	
	151.31	0.026	
$Ni_3(HITP)_2$	1.35	0.12	[9]
	2.33	0.12	
	4.65	0.12	
	9.34	0.11	

	13.98	0.11	
	23.46	0.11	
	32.84	0.11	
	46.71	0.10	
	69.39	0.09	
$Cu_3(HHTP)_2$	4.5	0.088	[10]
	9.3	0.084	
	14.1	0.081	
	23.3	0.077	
	32.9	0.073	
	46.6	0.068	
	70.2	0.066	
	93.1	0.060	
Covalent Organic Frameworks	0.75	0.027	[11]
	1.32	0.027	
	1.95	0.026	
	2.65	0.025	
	3.96	0.023	
	5.25	0.022	
	7.95	0.020	
	13.13	0.016	
	18.02	0.011	
	24.42	0.009	
$Ti_3C_2T_x$ MXene	0.5682	0.00947	[12]
	1.1244	0.00937	
	2.733	0.00911	
	5.244	0.00874	
	9.792	0.00816	

References

1 S. Funda, T. Ohki, Q. Liu, J. Hossain, Y. Ishimaru, K. Ueno, H. Shirai, *J. Appl. Phys.* 2016, **120**, 033103.

2 F. Ely, A. Matsumoto, B. Zoetebier, V. S. Peressinotto, M. K. Hirata, D. A. de Sousa, R. Maciel, *Org. Electron.* 2014, **15**, 1062.

3 L. Chen, C. Yuan, H. Dou, B. Gao, S. Chen, X. Zhang, *Electrochim. Acta* 2009, **54**, 2335.

4 D. Alemu, H.-Y. Wei, K.-C. Ho, C.-W. Chu, *Energy Environ. Sci.* 2012, **5**, 9662.

5 S. Garreau, G. Louarn, J. P. Buisson, G. Froyer, S. Lefrant, *Macromolecules* 1999, **32**, 6807.

6 S. Cho, M. Kim, J. Jang, *ACS Appl. Mater. Interfaces* 2015, **7**, 10213.

7 S. Nešpůrek, P. Kuberský, R. Polanský, M. Trchová, J. Šebera, V. Sychrovský, *Phys. Chem. Chem. Phys.* 2022, **24**, 541.

8 C. Zhang, T. M. Higgins, S.-H. Park, S. E. O'Brien, D. Long, J. N. Coleman, V. Nicolosi, *Nano Energy* 2016, **28**, 495.

9 W. Zhao, T. Chen, W. Wang, B. Jin, J. Peng, S. Bi, M. Jiang, S. Liu, Q. Zhao, W. Huang, *Sci. Bull.* 2020, **65**, 1803.

10 W. Zhao, T. Chen, W. Wang, S. Bi, M. Jiang, K. Y. Zhang, S. Liu, W. Huang, Q. Zhao, *Adv. Mater. Interfaces* 2021, **8**, 2100308.

11 W. Wang, W. Zhao, T. Chen, Y. Bai, H. Xu, M. Jiang, S. Liu, W. Huang, Q. Zhao, *Adv. Funct. Mater.* 2021, **31**, 2010306.

12 T. Guo, D. Zhou, S. Deng, M. Jafarpour, J. Avaro, A. Neels, J. Heier, C. Zhang, *ACS Nano* 2023, **17**, 3737.
